# Adrenocortical response during HUT test in patients with vasovagal syncope

**DOI:** 10.1007/s10286-026-01203-6

**Published:** 2026-03-18

**Authors:** Barbora Pavlovič Bačkorová, Zora Lazúrová, Monika Lukáčová, Kristína Valová, Peter Mitro, Ivica Lazúrová

**Affiliations:** 1 Department of Internal Medicine 1, Medical Faculty, P.J. Šafárik University Košice, Trieda SNP 1, 04011 Košice, Slovakia; 2 Department of Cardiology 1, Medical Faculty P.J. Šafárik University, Košice, Slovakia; 3 Department of Internal Medicine 4, Medical Faculty, P.J. Šafárik University, Košice, Slovakia

**Keywords:** Aldosterone, Cortisol, Head-up-tilt test, Vasovagal syncope

## Abstract

**Purpose:**

This prospective observational study was carried out to evaluate the response of adrenocortical hormones (cortisol and aldosterone) to head-up tilt (HUT)-induced vasovagal syncope in relation to hemodynamic changes.

**Subjects and methods:**

Blood samples from 79 patients (48 women and 31 men, age 19–73 years) who underwent the HUT test were analyzed for serum aldosterone and cortisol. On the basis of their response, patients were categorized into two groups: 50 HUT-positive (HUT+) and 29 HUT-negative (HUT−) patients.

**Results:**

HUT+ patients exhibited significantly lower baseline aldosterone levels compared with HUT− patients (203.1 ± 78.8 versus 266.2 ± 76.3 pg/ml, *p* = 0.0009). Similarly, aldosterone levels after the HUT test were lower in the HUT+ as compared with the HUT− group (249.5 ± 101.3 versus 351.4 ± 98.11 pg/ml, *p* < 0.0001). However, serum cortisol showed no significant differences between groups. Aldosterone increased in response to HUT in both groups. Aldosterone positively correlated with systolic and diastolic blood pressure, as well as heart rate. Integration of aldosterone with clinical assessment and additional laboratory tests may improve diagnostic accuracy and risk stratification.

**Conclusions:**

The results demonstrate significantly lower serum aldosterone concentrations in HUT+ patients at baseline and after HUT test in comparison with HUT− patients. Moreover, aldosterone levels positively correlated with systolic and diastolic blood pressure (BP). However, serum cortisol levels did not discriminate HUT+ from HUT− patients.

## Introduction

Syncope is characterized as a transient and brief loss of consciousness caused by a reduction in blood supply to the brain. Other conditions that manifest with similar symptoms must be excluded during the diagnostic process, including epileptic seizures, psychogenic causes, head trauma, or transient ischemic attacks [1]. The prevalence of experiencing at least one episode of syncope during a lifetime ranges from 30% to 50% in the general population [2]. Some authors have proposed that syncope may serve as a protective mechanism to prevent cerebral ischemia [3]. European Society of Cardiology (ESC) guidelines classify syncope into three categories: reflex (or neurally mediated), syncope due to orthostatic hypotension (including drug-induced, hypovolemia, and autonomic failure), and cardiac syncope (due to arrhythmias or structural heart damage).

Vasovagal syncope (VVS), the most common form of syncope, can be triggered by orthostatic factors (such as prolonged standing) or emotional stress (e.g., pain, the sight of blood) and is more common in younger individuals [1, 3]. Vasodepression results in reduced reflex activity, leading to vasodilation and inhibition of sympathetic vasoconstriction [2]. Low cardiac output due to reflex bradycardia results in cardioinhibitory syncope, with a predominance of parasympathetic nervous system activity. Diagnosis is established through medical history, physical examination, blood pressure measurement, electrocardiography, and laboratory analysis [1]. The global prevalence of VVS is approximately 16.4% (95% confidence interval (CI) 6–37.5%) [4].

Treatment modalities are still under investigation. Some strategies have shown effectiveness in reducing symptom recurrence, including patient education (avoiding trigger situations and recognizing prodromal symptoms), increasing fluid and salt intake, counterpressure maneuvers (such as handgrip or leg crossing), and pharmacological options including fludrocortisone (a mineralocorticoid agonist), midodrine (an alpha-adrenergic agonist), beta-blockers, pacing therapy, or cardioneuroablation [5, 6].

The role of the endocrine system in the pathogenesis of VVS remains unclear, and the sympathetic nervous system together with adenosine have been recognized as important participating agents [7–9]. However, the humoral response to orthostasis and its intolerance seems to be more complex, and research is focusing on various hormones and humoral systems, such as the adrenocorticotropic axis, renin–aldosterone system, or pituitary hormones [10–13].

The aim of the present study is to assess the changes in serum cortisol and aldosterone concentrations during vasovagal syncope induced by the head-up tilt test and to analyze their relation to the accompanying hemodynamic changes.

## Subjects and methods

This prospective observational study was conducted at the Syncope Unit of the 1st Department of Cardiology, Medical Faculty, P. J. Šafárik University, Košice, Slovakia. Data were collected from February 2024 to February 2025. The study was approved by the local ethics committee. A total of 79 patients with history of recurrent syncope were included. All patients underwent a diagnostic head-up tilt test (HUT). Of these, 29 patients demonstrated a normal response during the test (i.e., did not develop syncope symptoms) and were classified as the HUT negative (HUT−) group. The remaining (HUT+) patients were categorized according to the VAsovagal Syncope International Study (VASIS) classification [14–16]. Exclusion criteria included presence of structural heart disease and use of interfering medications (including angiotensin-converting enzyme inhibitors, angiotensin receptor and neprilysin inhibitors, oral contraceptives, mineralocorticoid receptor antagonists, corticosteroids, antidepressants, antipsychotics, metoclopramide, proton-pump inhibitors, and dopamine agonists). Additional exclusion criteria were pregnancy, breastfeeding, anemia, liver cirrhosis, oncological and neurological diseases, as well as untreated endocrinopathies. Patients were instructed to follow specific regimen restrictions prior to the test, including fasting, refraining from physical activity on the day of testing, and avoiding smoking, coffee, green or black tea, alcohol, and other caffeine-containing beverages.

The head-up tilt (HUT) test was performed using the Italian protocol in the morning: patients rested in supine position for 5 min, followed by tilting to a 60° angle for 20 min (passive phase). If no symptoms occurred during this phase, it was followed by a provocation phase involving the sublingual administration of nitroglycerin and an additional 15 min in the tilted position [2, 17]. Patients were continuously monitored using electrocardiography (ECG), heart rate monitoring, and electroencephalography (EEG). Brachial blood pressure was measured every 2 min using an automated oscillometric manometer. Peripheral venous blood samples were collected before the HUT test (in supine position) and immediately after the occurrence of syncope. In cases where vasovagal symptoms did not occur, blood samples were taken immediately after the HUT test. The samples were centrifuged and stored at −70 °C until laboratory analysis. Aldosterone levels were measured using radioimmunoassay (Beckman Coulter, Prague, Czech Republic; lowest reportable value 25.77 pg/mL; inter-assay coefficient of variation (CV) ≤ 15.26%; intra-assay CV ≤ 23.96%). Cortisol levels were also measured using radioimmunoassay (Beckman Coulter, Prague, Czech Republic; lowest reportable value: 8.6 nmol/L; dynamic range: 8.6 to 2000 nmol/L; inter-assay CV: 6.2–18.5%; intra-assay CV ≤ 13.7%).

Statistical analysis and graphical visualizations were performed using GraphPad Prism and Python software. Data are presented as mean ± standard deviation. Normality of distribution was assessed using the Shapiro–Wilk test. Categorical variables were analyzed using Pearson’s chi-squared test. Continuous variables were compared using Student’s *t*-test or Mann–Whitney test for unpaired data, and the Wilcoxon test for paired samples. Group comparisons were performed using one-way analysis of variance (ANOVA). For aldosterone, the sample size provided > 80% power to detect a difference of 100 pg/mL. In contrast, the same sample provided only ~ 9% power to detect a difference of 30 pg/mL for cortisol, indicating limited sensitivity for this hormone. *p*-Value below 0.05 was considered statistically significant.

## Results

We enrolled 79 patients (aged 19–73 years) with suspected vasovagal syncope, including 48 female and 31 male participants. Mean baseline hemodynamic and humoral parameters in the HUT + and HUT− groups are presented in Table 1. There were no significant differences between the two groups in their baseline characteristics or hemodynamic parameters (Table 1), except for heart rate, which was significantly higher in the HUT+ group (76.80 ± 13.10 versus 70.14 ± 14.16 bpm, *p* = 0.013). Among the hormonal parameters, significantly lower aldosterone levels were observed in the HUT+ group before the HUT test compared with the HUT− group (203.1 ± 78.8 versus 266.2 ± 76.34 pg/mL, *p* = < 0.001). Cortisol levels did not differ significantly between the groups. Of patients, 12% had a positive HUT test during the passive phase, while the remaining 44 individuals required nitroglycerin administration to confirm the diagnosis.

**Table 1:** Baseline hemodynamic and hormonal characteristics in HUT+ and HUT− patients

	HUT+ group (*n* = 50)	HUT− group (*n* = 29)	*p*-Value
Male/female	17 (34%)/33 (66%)	14 (48.3%)/15 (51.7%)	0.239
Age (years)	42.84 ± 16.83	44.9 ± 13.57	0.535
Baseline SBP (mmHg)	117.5 ± 19.64	119.0 ± 15.52	0.507
Baseline DBP (mmHg)	77.76 ± 11.59	76.00 ± 12.29	0.5
Baseline HR (bpm)	76.80 ± 13.10	70.14 ± 14.16	0.013*
Cortisol (nmol/L)	362.7 ± 174.6	365.1 ± 195.7	0.764
Aldosterone (pg/mL)	203.1 ± 78.8	266.2 ± 76.34	< 0.001***

*SBP* systolic blood pressure, *DBP* diastolic blood pressure, *HR* heart rate, *HUT+* HUT positive, *HUT−* HUT negative

*p ≤ 0.05, ***p ≤ 0.001

To assess the potential influence of nitroglycerin, a sensitivity analysis was performed comparing patients with syncope during the passive phase, patients with syncope after nitroglycerin administration, and HUT− individuals. Baseline aldosterone levels differed significantly among the three groups (*p* = 0.0016).

In response to the HUT test (Table 2), systolic blood pressure, diastolic blood pressure, and heart rate significantly decreased in the HUT+ group (*p* < 0.0001), as expected. Similar to baseline values, significantly lower aldosterone levels were observed in the HUT+ group after syncope (249.5 ± 101.3 versus 351.4 ± 98.11 pg/mL, *p* < 0.0001). Δ aldosterone, i.e., aldosterone after − aldosterone before the HUT test, was also significantly lower in the HUT+ group (45.82 ± 65.00 versus 84.83 ± 58.97 pg/mL, *p* < 0.01). After the HUT test, patients with a positive response were classified as follows according to the VASIS classification: 31 patients as VASIS I (62%), 2 patients as VASIS IIa (4%), 7 patients as VASIS IIb (14%), and 10 patients as VASIS III (20%), respectively. Analysis of hormonal levels across different VASIS subtypes revealed no statistically significant differences between the groups (Table 3).

**Table 2:** Hemodynamic and humoral changes in HUT+ and HUT− groups after the HUT test

	HUT+ group (*n* = 50)	HUT− group (*n* = 29)	*p*-Value
SBP (mmHg)	73.60 ± 19.85	117.1 ± 15.15	< 0.0001****
DBP (mmHg)	39.20 ± 25.12	76.31 ± 10.20	< 0.0001****
HR (bpm)	56.76 ± 34.16	73.69 ± 10.35	< 0.001***
Cortisol (nmol/L)	379.1 ± 165.8	410.6 ± 183.0	0.462
Aldosterone (pg/mL)	249.5 ± 101.3	351.4 ± 98.11	< 0.0001****
Δ Aldosterone (pg/mL)	45.82 ± 65.00	84.83 ± 58.97	< 0.01**
Δ Cortisol (nmol/L)	14.00 ± 141.1	48.53 ± 138.4	0.291

*SBP* systolic blood pressure, *DBP* diastolic blood pressure, *HUT +* HUT positive, *HUT−*HUT negative, *Δ aldosterone* aldosterone after − aldosterone before the HUT test

**p ≤ 0.01, ***p ≤ 0.001, ****p ≤ 0.0001

**Table 3:** Hormonal levels among VASIS subtypes

	VASIS I (*n* = 31)	VASIS II (*n* = 9)	VASIS III (*n* = 10)	*p*-Value
Cortisol baseline (nmol/L)	359.6 ± 151.7	416.9 ± 286.4	323.5 ± 108.0	0.993
Cortisol HUT (nmol/L)	387.1 ± 165.4	383.6 ± 195.3	350.4 ± 153.0	0.762
Aldosterone baseline (pg/mL)	197.5 ± 85.53	182.3 ± 74.32	239.3 ± 50.17	0.075
Aldosterone HUT (pg/mL)	242.3 ± 106.1	233.0 ± 117.1	286.5 ± 64.34	0.144

*HUT* head-up tilt

**Fig. 1:**
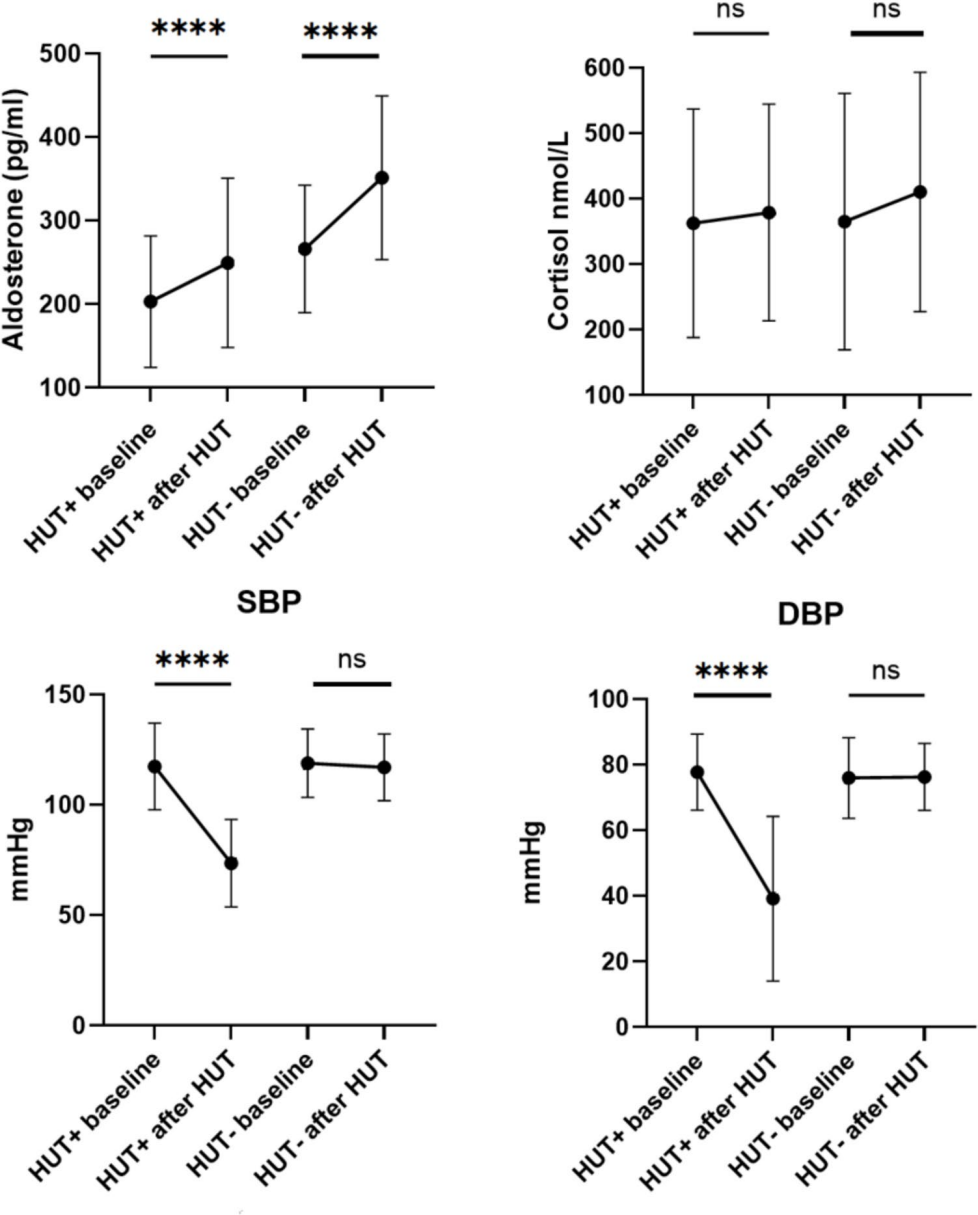
Hemodynamic and hormonal changes during HUT test. *SBP* systolic blood pressure, *DBP* diastolic blood pressure, *HUT+* HUT positive, *HUT−* HUT negative ****p ≤ 0.0001, ns (nonsignificant),

Analyzing the dynamic changes in hormonal levels before and after the HUT test (Fig. 1), we observed a significant increase in aldosterone in both the HUT+ (*p* ≤ 0.0001) and the HUT− group (*p* ≤ 0.0001). Despite this increase, the peak aldosterone levels in the HUT+ group did not reach the baseline levels observed in the HUT− group. Both groups showed a nonsignificant increase in cortisol levels in response to the HUT test. As expected, systolic and diastolic blood pressure significantly decreased in the HUT+ group, whereas no significant changes in hemodynamic parameters were observed in the HUT− group.

**Fig. 2:**
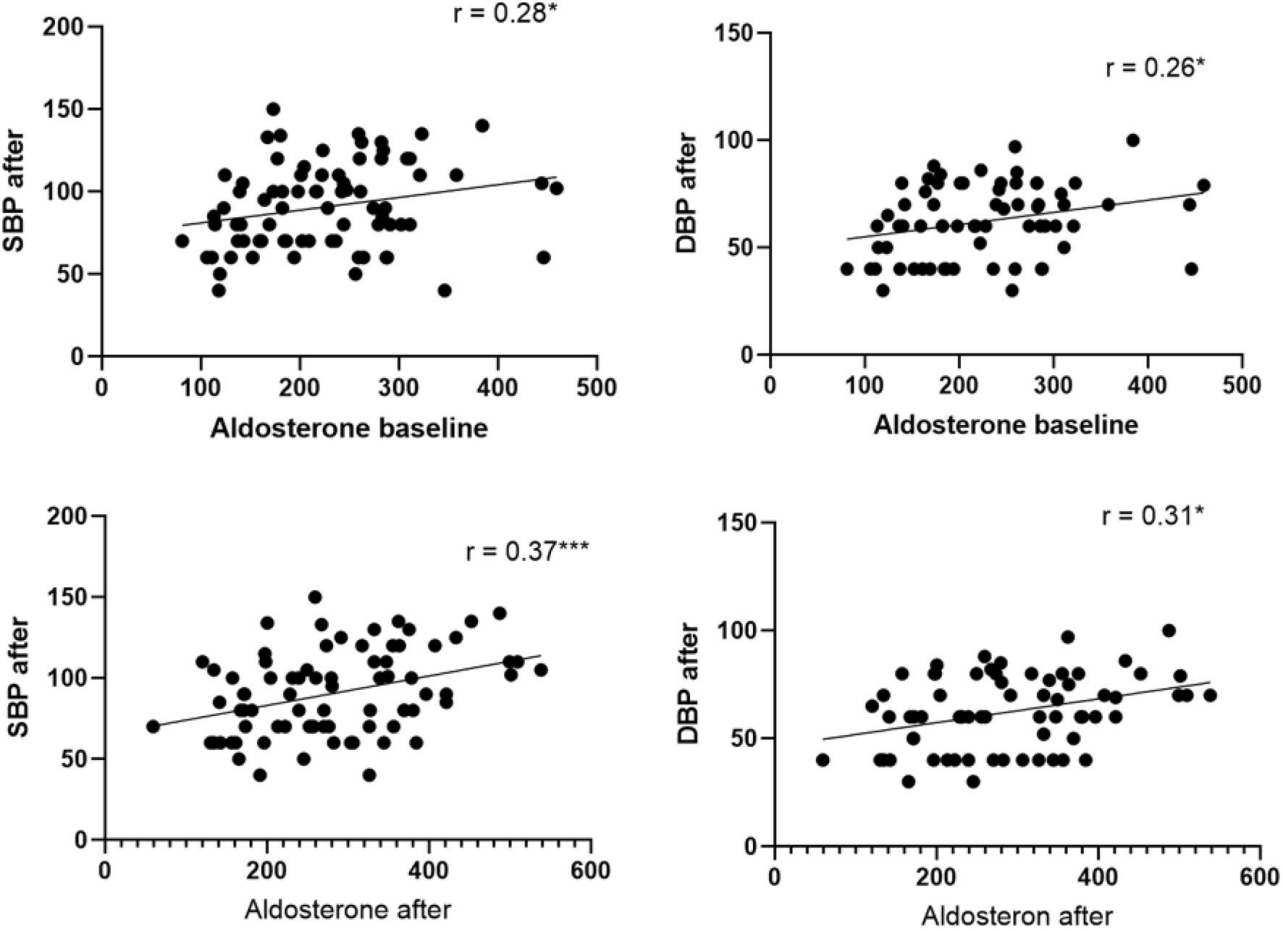
Correlation between hemodynamic parameters and hormones. *SBP* systolic blood pressure, *DBP* diastolic blood pressure, *p ≤ 0.05, ***p ≤ 0.001

Baseline aldosterone levels were positively correlated with post-HUT systolic and diastolic blood pressure (Fig. 2) (*r* = 0.28, *p* = 0.02 and *r* = 0.26, *p* = 0.03). A positive correlation was also observed between post-HUT aldosterone levels and systolic and diastolic blood pressure (*r* = 0.37, *p* = < 0.001 and *r* = 0.31, *p* = 0.01). There was no significant correlation between baseline SBP or DBP and either baseline (*r* = –0.09, *p* = 0.422 and *r* = –0.03, *p* = 0.761) or post-HUT aldosterone levels (*r* = –0.01, *p* = 0.951 and *r* = 0.06, *p* = 0.63). Baseline and post-HUT aldosterone correlated with postsyncopal heart rate (*r* = 0.28, *p* = 0.02 and *r* = 0.27, *p* = 0.02). In contrast, cortisol levels were positively associated only with baseline heart rate (*r* = 0.26, *p* = 0.041).

**Fig. 3:**
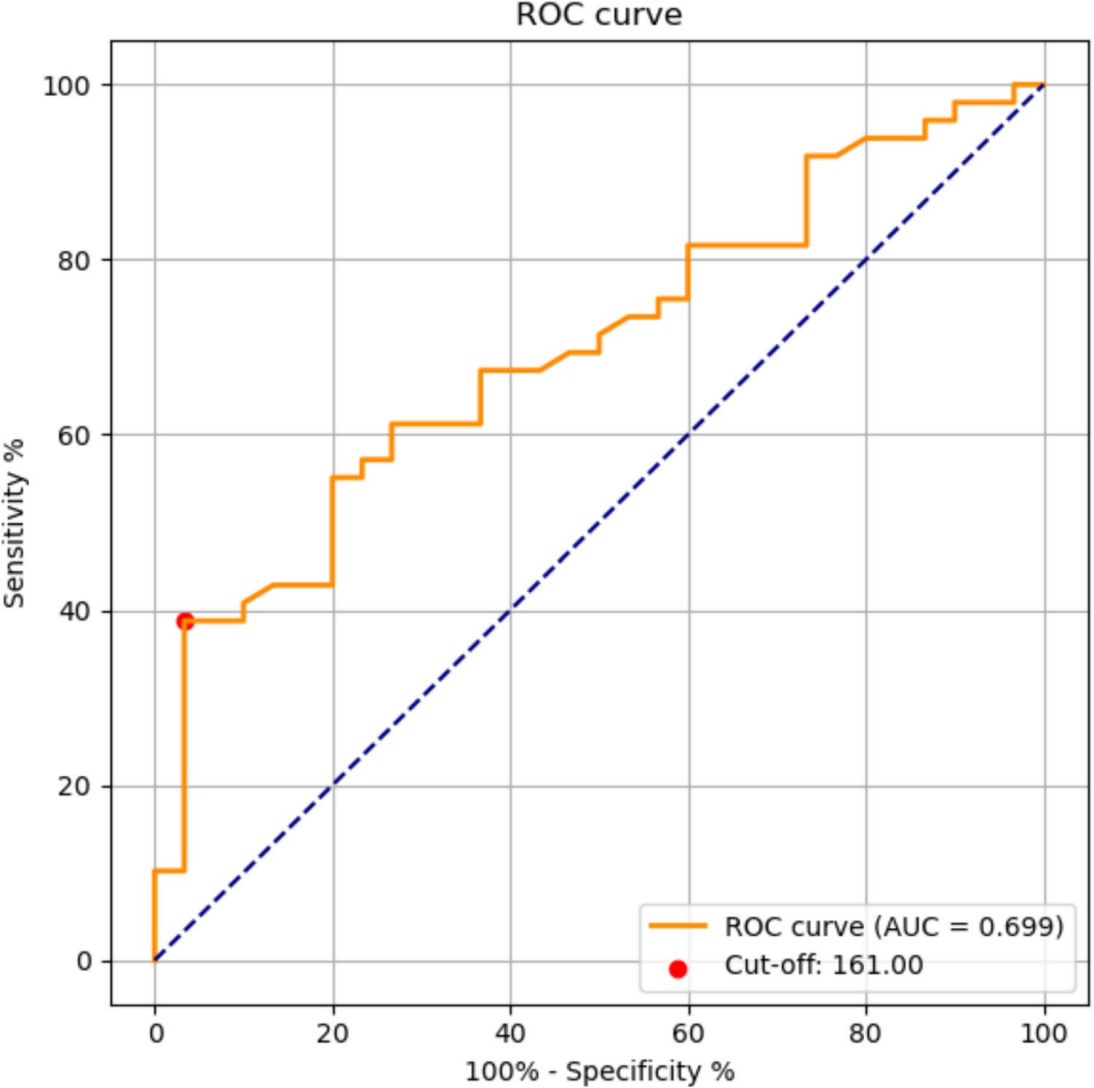
ROC curve

According to the receiver operating characteristic (ROC) curve analysis (Fig. 3), the optimal aldosterone cutoff value to distinguish between HUT+ and HUT− was 161 pg/mL, yielding a low sensitivity of 39% (95% CI 23.3–50.0%) but very high specificity of 97% (95% CI 88.9–100.0%). Aldosterone levels below this threshold were associated with an 18.37-fold increased likelihood of positive HUT test (odds ratio (OR) ~18; 95% CI 2.3–146; *p* = < 0.001, Fisher’s exact test). The overall discriminatory ability of aldosterone was intermediate (area under the curve (AUC) = 0.699; 95% CI 0.565–0.804).

## Discussion

The aim of this study was to assess the response of serum cortisol and aldosterone during the HUT test in HUT+ patients and to compare it with the response in HUT− subjects. Hormonal evaluation revealed significantly lower baseline and post-tilt serum aldosterone concentrations in the HUT+ compared with HUT− group. Analysis of the hormonal response showed a significant increase in serum aldosterone concentration during the HUT test in both HUT+ and HUT− subjects, but the serum aldosterone concentration after HUT in HUT+ participants did not reach the values observed in the HUT− group. In contrast to aldosterone, serum cortisol levels did not differ between groups, and also did not change significantly in either the HUT+ or HUT− group. The observed differences in aldosterone are likely reliable, given the adequate statistical power of the study.

The renin–angiotensin–aldosterone (RAA) axis responds to orthostatic changes, with hypovolemia stimulating this system. Gravitational forces after standing lead to accumulation of body fluid in the abdomen and legs, resulting in decreased venous return to the heart and a drop in blood pressure [18]. It is generally expected that syncope due to hypovolemia would increase aldosterone levels. Surprisingly, our data show lower aldosterone levels in patients diagnosed with vasovagal syncope compared with HUT− participants. These results indicate that the observed association between aldosterone levels and syncope is not explained by age alone or by nitroglycerin administration.

After the HUT test, aldosterone levels increased significantly in both groups, leading to the hypothesis that hypovolemia during the test may activate the renin–angiotensin–aldosterone system (RAAS). However, HUT+ patients may have a chronically activated and altered RAAS, leading to downregulation and lower baseline activity, as their peak aldosterone levels did not reach the baseline levels observed in the HUT− group. Ray et al. reported low aldosterone in patients with postural orthostatic tachycardia syndrome (POTS), who exhibited low aldosterone levels despite hypovolemia. The authors termed this phenomenon the “renin–aldosterone paradox,” in which hypovolemia fails to activate the RAAS or stimulate aldosterone secretion [19]. Vanderheyden and colleagues observed higher aldosterone levels in patients with vasodepressor-type syncope and in those without syncope during the HUT test. Conversely, individuals with cardioinhibitory syncope demonstrated elevated norepinephrine but a blunted aldosterone response, suggesting that endocrine reactions may vary among syncope subtypes [20]. Similarly, we found a trend toward lower aldosterone levels in patients with cardioinhibitory and mixed types of VVS, when compared with vasodepressor VVS, but the results did not reach statistical significance.

In an earlier study, Gajek et al. (2005) also analyzed aldosterone and renin levels during the HUT test in patients with neurally mediated syncope. They reported increased plasma renin activity 10 min after HUT and a significant rise in aldosterone immediately after the test, which aligns with our results [11]. On the other hand, a different study found higher aldosterone levels in HUT+ children, possibly as a compensatory mechanism for impaired sympathetically mediated vasoconstriction [21]. An earlier study reported no significant differences in baseline hormone levels; however, aldosterone increased in symptomatic patients after tilt but remained unchanged in nonsyncopal individuals [22]. In contrast to those findings and ours, a cohort study by Yamanouchi, Shehadeh, and Fouad-Tarazi (1998) found no significant differences in aldosterone levels between HUT+ and HUT− patients [23].

Current data suggest that the hypothalamic–pituitary–adrenal (HPA) axis may play a role in the pathogenesis of vasovagal syncope. Cortisol secretion is stimulated in response to both acute and chronic stress. VVS induced by venepuncture has been associated with elevated cortisol levels, and patients with higher baseline cortisol were more likely to experience a vasovagal reaction [24, 25]. Another study analyzing cortisol response to the HUT test found similar baseline cortisol levels between HUT+ and HUT− participants, but cortisol increased more significantly post-HUT in the HUT+ group [26]. Some studies have reported significantly higher cortisol levels in patients with vasovagal syncope compared with healthy subjects [25, 27]. Our findings, however, did not demonstrate significant differences in cortisol levels between the HUT+ and HUT− groups. Although a mild increase in cortisol was observed after the HUT test in both groups, this change was not statistically significant.

Cortisol, as well as prolactin, may reflect changes in central serotonergic activity, which could play a role in vasovagal syncope. Serotonin may influence vagal afferent inhibition of renal nerve activity during orthostasis [28]. One study found increased cortisol levels in patients with syncope after the HUT test. Since cortisol secretion is influenced also by serotonergic activity, the authors proposed a theory of increased central serotonergic activation. Activation of the serotonergic system results in hypotension through sympathetic inhibition and the release of hormones such as prolactin and adrenocorticotropic hormone (ACTH) [29]. The lack of higher cortisol levels in the VVS group in our study may reflect the physiological stress induced by the HUT test itself, as perceived by healthy individuals.

Aldosterone levels below 161 pg/mL were strongly associated with HUT+ status, yielding a sensitivity of 39% and a specificity of 97%. While this cutoff helps to identify individuals at risk, its moderate overall discriminatory ability limits its use as a standalone tool. However, individuals with higher aldosterone levels are less likely to develop syncope, suggesting that elevated aldosterone may help to exclude susceptibility to syncope rather than to predict its occurrence. Integration with clinical assessment and additional laboratory tests may improve the diagnostic accuracy and risk stratification. The wide confidence interval for the odds ratio and low sensitivity highlight the need for larger cohorts and further studies to confirm these findings. Such an analysis has not been published in the literature so far.

Aldosterone, as a mineralocorticoid, is well known for its role in maintaining the extracellular fluid volume [30]. During syncope, gravitational forces cause accumulation of body fluids in the lower extremities, resulting in hypovolemia and hypotension [31]. Owing to the observational nature of the study, our findings demonstrate an association between altered aldosterone dynamics and susceptibility to vasovagal syncope. Elevated aldosterone may represent an effective compensatory response to orthostatic stress, potentially preventing syncope and helping to stabilize blood pressure and heart rate, although we cannot establish a causal role of aldosterone in the pathogenesis of vasovagal syncope.

Overall, this study demonstrates lower serum aldosterone levels at baseline and after the HUT test in HUT+ compared with HUT− participants, which represents the main finding. To the best of our knowledge, this is the first study to report these findings in the largest cohort to date, and it may suggests potential therapeutic relevance for the use of mineralocorticoids in treating VVS patients; however, further studies analyzing aldosterone as well as renin are needed to confirm this hypothesis.

This study has several limitations. One is the relatively small sample size; however, it still allows for relevant statistical evaluation. Additionally, we did not measure plasma renin activity or concentration, or potassium levels. Although nitroglycerin administration as part of a standardized protocol does not directly affect the RAAS, its vasodilatory effects may have influenced aldosterone levels. Plasma renin activity is less stable and can be influenced by multiple uncontrollable factors. Moreover, we did not consider women’s menstrual status in our analysis. Further studies investigating the renin–angiotensin–aldosterone system in VVS patients are warranted, especially regarding the potential use of mineralocorticoids.

## Conclusions

This study demonstrates significantly lower serum aldosterone levels at baseline and after the HUT test in HUT+ compared with HUT− participants. Although serum aldosterone increased after tilt in both HUT+ and HUT− groups, the aldosterone response in the HUT+ group did not reach the baseline levels observed in the HUT− group. Better understanding of this phenomenon may lead to improved treatment and diagnostic strategies and help identify patients who could benefit from mineralocorticoid therapy. Establishing an aldosterone threshold could serve as one of the tools for differentiating patients and guiding potential pharmacological intervention with fludrocortisone.

## Data Availability

The datasets generated and/or analyzed during the current study are available from the corresponding author on reasonable request.
